# Effect, safety, timing and dose of thoracic radiotherapy plus third-generation EGFR-TKIs as first-line treatment in patients with EGFR-mutated oligo-organ metastatic NSCLC

**DOI:** 10.3389/fonc.2025.1707876

**Published:** 2025-11-26

**Authors:** Xinhang Gu, Jiaxiao Geng, Hongfu Sun, Qiang Cao, Yan Yi, Baosheng Li

**Affiliations:** Department of Radiation Oncology, Shandong Cancer Hospital and Institute, Shandong First Medical University and Shandong Academy of Medical Sciences, Jinan, Shandong, China

**Keywords:** non-small cell lung cancer, radiotherapy, epidermal growth factor receptor, tyrosine kinase inhibitor, radiation dosage

## Abstract

**Background:**

Epidermal growth factor receptor (EGFR) tyrosine kinase inhibitor (TKI)-based combination therapy modalities for patients with EGFR-mutated stage IV non-small cell lung cancer (NSCLC) are being investigated. We evaluated the value and safety of third-generation EGFR-TKIs combined with thoracic radiotherapy (TRT) in patients with oligo-organ metastatic, along with the optimal TRT timing and dose.

**Methods:**

We retrospectively enrolled patients with EGFR-mutated oligo-organ metastatic NSCLC who received first-line third-generation EGFR-TKIs from 2018 to 2023. Patients were divided into TKI-alone and TKI+TRT groups according to whether TRT was added. Propensity score matching (PSM) was implemented to decrease bias. The primary endpoint was progression-free survival (PFS). Secondary endpoints included overall survival (OS) and safety.

**Results:**

A total of 236 patients were included, the median follow-up was 28.4 months. After PSM, baseline characteristics were balanced between the TKI+TRT (n=69) and TKI-alone (n=108) groups. Compared with the TKI-alone group, the TKI+TRT group presented significantly better PFS (28.6 vs. 19.8 months, hazard ratio [HR]=0.48, P = 0.00024) and OS (42.2 vs. 35.1 months, HR = 0.54, P = 0.039). In patients who received TRT, the tumor shrinkage group demonstrated significantly improved PFS (HR = 0.36, P = 0.0035) and OS (HR = 0.13, P = 0.0012) compared to the tumor enlargement/Stabilization group. The high-dose group showed superior PFS (HR = 0.43, P = 0.011) and OS (HR = 0.36, P = 0.023) compared to the low-dose group. Only 5.8% of patients in the TKI+TRT group developed grade ≥3 pneumonitis.

**Conclusion:**

Incorporating TRT provided significant survival benefits in patients with oligo-organ metastatic NSCLC who received first-line third-generation EGFR TKIs, with acceptable side effects. The administration of higher radiation doses during a phase of tumor shrinkage may be associated with optimal outcomes.

## 1 Introduction

Lung cancer has the highest incidence and mortality rates among all cancer types. Approximately 80–85% of lung cancer cases are diagnosed as non-small cell lung cancer (NSCLC) (1, 2). Moreover, 30%–50% and 20% of NSCLC patients in Asia and Caucasus, respectively, have a mutation in the epidermal growth factor receptor (EGFR) gene (3). During NSCLC treatment, EGFR mutations have been identified as a critical therapeutic target.

EGFR tyrosine kinase inhibitors (TKIs) have become a first-line therapy option for patients with stage IV NSCLC (4) due to their notable antitumor activity and extended progression-free survival (PFS) and overall survival (OS). However, numerous patients exhibit disease progression, with the lung as the most typical site of initial progression; specifically, 45% of patients exhibit progression at this site (5). Therefore, the search for combined treatment strategies to further improve efficacy and prolong survival has become a focus of research.

As a local treatment, thoracic radiotherapy (TRT) has been shown to be effective in controlling local tumor growth and relieving symptoms, with approximately 70% of lung cancer patients receiving such therapy (6). Current consensus defines oligometastasis (≤3 metastatic organs with ≤5 metastatic sites) as a distinct subset of stage IV NSCLC that may benefit from local therapy. TRT integrated with chemotherapy or targeted treatment improves survival among patients with oligometastatic NSCLC. As indicated by a phase II clinical trial, in patients with oligometastatic NSCLC, survival was improved by treatment with chemotherapy or targeted therapy followed by TRT compared to treatment with maintenance therapy, showing a median OS of 41.2 vs. 17.0 months and a median PFS of 11.9 vs. 3.9 months (7). The SINDAS trial showed that in EGFR-mutant oligometastatic NSCLC, combining RT with first-line first-generation TKI significantly improved PFS and OS over TKI alone (8).

Slightly different from the aforementioned study, in our previous prospective study, we found that TRT combined with first-generation EGFR-TKI resulted in a PFS of 17.1 vs. 10.6 months and an OS of 34.4 vs. 26.2 months compared to TKI alone in patients with EGFR-mutated oligo-organ metastatic (≤3 metastatic organs, metastatic sites unrestricted) NSCLC, confirming the clinical benefit of TRT in oligo-organ metastatic population (9).Third-generation EGFR TKIs, such as osimertinib (a global standard), and the domestically developed agents in China, almonertinib and furmonertinib, exhibit enhanced selectivity and diminished off-target effects against EGFR mutations compared to first-generation TKIs, as well as greater efficacy against brain metastases (10–12). Almonertinib and furmonertinib have been approved in China and demonstrated superior PFS over first-generation TKIs in phase III trials. Therefore, it may be posited that combining third-generation EGFR TKIs with TRT represents a more efficient therapy strategy for patients with oligo-organ metastatic NSCLC. However, several important questions remain to be answered. First, does the high efficacy of third-generation TKIs reduce the benefits of TRT or, conversely, lead to improved survival outcomes? Second, which factors influence the survival outcomes of combined treatment modalities, and what are the optimal timing and doses for TRT? Finally, will adverse effects increase, and are they tolerable? Therefore, we conducted a retrospective study to address these issues and provide evidence for clinical decision-making.

## 2 Materials and methods

### 2.1 Patients

In total, 614 patients with EGFR-mutated stage IV NSCLC who received third-generation EGFR-TKIs with or without TRT from January 2018 to December 2023 at the Shandong Cancer Hospital were retrospectively reviewed. The inclusion criteria were (i) verified NSCLC with EGFR exon 19 deletions or exon 21 mutations, (ii) clinical stage IV(AJCC eight edition)and (iii) the initiation of a first-line therapy with third-generation EGFR-TKIs. The exclusion criteria were (i) primary resistance, (ii) combination with other anticancer drugs, (iii) another active cancer, (iv) Eastern Cooperative Oncology Group performance status (ECOG PS) ≥2, (v) the number of metastatic organs>3, and (vi) incomplete clinical data. Patients were divided into TKI-alone and TKI+TRT groups according to whether TRT was added. This study received approval from the Ethical Review Committee of Shandong Cancer Hospital. Given that this was a retrospective study, patient informed consent was not required.

### 2.2 TKI treatment

In all cases, a multidisciplinary team (MDT) was responsible for disease evaluation and treatment regimen selection, with EGFR-TKIs being the preferred treatment. The EGFR-TKIs administered to the enrolled patients were exclusively third-generation EGFR-TKIs: osimertinib (80 mg/dose, qd, oral), almonertinib (110 mg/dose, qd, oral), or furmonertinib (80 mg/dose, qd, oral).

### 2.3 Radiotherapy

The decision regarding TRT was made by a MDT after a comprehensive assessment of the patient’s individual clinical circumstances, tumor biology, and preferences. Patients in the TKI+TRT group received both treatments concurrently, with TRT comprising stereotactic body radiation therapy (SBRT) or intensity-modulated radiotherapy (IMRT). The gross tumor volume (GTV) was outlined in an attempt to encompass the primary lung tumors and positive regional lymph nodes. The planning target volume was the GTV plus a 5-10-mm margin.

The biologically effective dose (BED) was determined using the linear-quadratic formula (1) for standardized physical dose values (13):

(1) BED = n×d[(1 + d/(α/β))]

Here, n indicates the number of treatment fractions, d indicates the dose per fraction, α and β indicate the linear and quadratic coefficients, respectively, and α/β is set to 10 Gy. Organs at risk were protected by radiation plan optimization. Radiation therapy was not mandatory at sites of distant metastasis, such as the brain and bones.

### 2.4 Follow-up

All enrolled patients underwent regular follow-up assessments, which included enhanced computed tomography (CT), magnetic resonance imaging, whole-body bone imaging, and positron emission tomography/CT. All patients had follow-up data until the cutoff date (September 5, 2024) or death. The primary endpoints was progression-free survival (PFS).Secondary endpoints included overall survival (OS) and safety. PFS was measured from the beginning of EGFR-TKI therapy to the point of objective death or disease development. OS was defined as the duration from the start of EGFR-TKI treatment to death. The Common Terminology Criteria for Adverse Events (v 5.0) was employed to appraise treatment-related adverse events (TRAEs).

### 2.5 Statistical analysis

Propensity score matching (PSM) was adopted to formulate balanced cohorts, with matching variables including sex, age, ECOG PS, smoking history, T stage, N stage, number of metastatic organs, number of metastatic sites, TNM stage, brain metastasis status, EGFR mutation type, and TKI regimen (14). A caliper value of 0.2 was utilized to achieve 1:2 matching between the two groups. The chi-squared test and Fisher’s exact test were used to contrast clinical features and TRAEs between both groups. The Kaplan–Meier survival curve was used to measure the survival rate. The log-rank test was adopted to appraise significance. The Cox proportional hazard model was used to determine the hazard ratio (HR). In addition, data were analyzed using R software (v 4.3) and SPSS software (v 27.0). P<0.05 was considered statistically significant.

## 3 Results

### 3.1 Patient population

In total, 236 patients with EGFR-mutated oligo-organ metastatic NSCLC were included in our study, with 82 treated with TKI+TRT and 154 treated with TKI alone (**Supplementary Figure 1**). Their median age reached 58 years (range, 36–78 years) in the TKI+TRT group and 62 years (range, 32–84 years) in the TKI-alone group.

At baseline, there was an imbalance in the clinical characteristics, including sex, ECOG PS, number of metastatic organs, and brain metastasis between the two groups. Therefore, we performed 1:2 PSM to mitigate the imbalance of baseline levels between the two arms, resulting in 69 patients in the TKI+TRT group and 108 in the TKI-alone group. After PSM, the patients’ clinical characteristics were balanced. **Table 1** summarizes the baseline patient features before and after PSM. After PSM, in the TKI-alone group, 49 (45.4%) patients were diagnosed with brain metastasis, and 59 (54.6%) were diagnosed with bone metastasis. In contrast, in the TKI+TRT group, the respective counts were 27 (39.1%) and 36 (52.2%). **Supplementary Table 1** lists the metastatic organs.

**Table 1:** Baseline patient characteristics before and after PSM.

Characteristics		Before PSM				After PSM		
	TKI alone(n=154,%)	TKI+TRT(n=82,%)	P value	SMD	TKI alone(n=108,%)	TKI+TRT(n=69,%)	P value	SMD
Sex			0.006	0.395			0.407	0.152
Male	46 (29.9%)	40 (48.8%)			42 (38.9%)	32 (46.4%)		
Female	108 (70.1%)	42 (51.2%)			66 (61.1%)	37 (53.6%)		
Age			0.123	0.231			0.869	0.049
≤60	65 (42.2%)	44 (53.7%)			49 (45.4%)	33 (47.8%)		
>60	89 (57.8%)	38 (46.3%)			59 (54.6%)	36 (52.2%)		
ECOG PS			0.001	0.503			0.405	0.153
0	62 (40.3%)	53 (64.6%)			56 (51.9%)	41 (59.4%)		
1	92 (59.7%)	29 (35.4%)			52 (48.1%)	28 (40.6%)		
Smoking			0.796	0.058			0.791	0.068
NO	122 (79.2%)	63 (76.8%)			83 (76.8%)	51 (73.9%)		
YES	32 (20.8%)	19 (23.2%)			25 (23.2%)	18 (26.1%)		
T stage			0.746	0.153			0.768	0.166
T1	43 (27.9%)	18 (22.0%)			29 (26.9%)	14 (20.3%)		
T2	68 (44.2%)	38 (46.3%)			50 (46.3%)	33 (47.8%)		
T3	17 (11.0%)	9 (11.0%)			12 (11.1%)	9 (13.1%)		
T4	26 (16.9%)	17 (20.7%)			17 (15.7%)	13 (18.8%)		
N stage			0.927	0.037			0.737	0.085
N0	26 (16.9%)	15 (18.3%)			19 (17.6%)	10 (14.5%)		
N+	128 (83.1%)	67 (81.7%)			89 (82.4%)	59 (85.5%)		
No. of metastatic organs			0.047	0.353			0.540	0.171
1	57 (37.0%)	39 (47.5%)			50 (46.3%)	30 (43.5%)		
2	48 (31.2%)	29 (35.4%)			31 (28.7%)	25 (36.2%)		
3	49 (31.8%)	14 (17.1%)			27 (25.0%)	14 (20.3%)		
No. of metastatic sites			0.674	0.077			0.642	0.097
1-5	90 (58.4%)	51 (62.2%)			67 (62.0%)	46 (66.7%)		
>5	64 (41.6%)	31 (37.8%)			41 (38.0%)	23 (33.3%)		
TNM			0.090	0.250			0.880	0.049
IV A	37 (24.0%)	29 (35.4%)			32 (29.6%)	22 (31.9%)		
IV B	117 (76.0%)	53 (64.6%)			76 (70.4%)	47 (68.1%)		
Brain metastasis			0.010	0.380			0.508	0.127
NO	69 (44.8%)	52 (63.4%)			59 (54.6%)	42 (60.9%)		
YES	85 (55.2%)	30 (36.6%)			49 (45.4%)	27 (39.1%)		
EGFR mutation type			0.928	0.031			0.935	0.037
Exon 19 deletion	84 (54.6%)	46 (56.1%)			63 (58.3%)	39 (56.5%)		
Exon 21 mutation	70 (45.4%)	36 (43.9%)			45 (41.7%)	30 (43.5%)		
TKI			0.634	0.130			0.849	0.086
Osimertinib	94 (61.0%)	45 (54.9%)			66 (61.1%)	41 (59.4%)		
Almonertinib	56 (36.4%)	34 (41.5%)			39 (36.1%)	25 (36.2%)		
Furmonertinib	4 (2.6%)	3 (3.6%)			3 (2.8%)	3 (4.4%)		
Response evaluation*			0.861	0.044			0.971	0.031
PR	102 (66.2%)	56 (68.3%)			72 (66.7%)	47 (68.1%)		
SD	52 (33.8%)	26 (31.7%)			36 (33.3%)	22 (31.9%)		

PSM, propensity score matching; ECOG PS, Eastern Cooperative Oncology Group performance status; EGFR, epidermal growth factor receptor; TKI, tyrosine kinase inhibitor; TRT, thoracic radiotherapy.

In the TKI+TRT group before PSM, the TRT dose range was 30–75 Gy. The median BED of TRT was 67.1 Gy (36.0–112.5 Gy) (Equation 1). The median PTV volume was 101.8 cm^3^.The radiotherapy sites and dose during initial treatment between the two groups after PSM are provided in **Supplementary Table 2**. In the TKI+TRT group after PSM, all patients received TRT with a BED of ≥58.5 Gy. In the TKI-alone group, radiotherapy was provided to 15 patients (13.9%) for bone metastases and 21 patients (19.4%) for brain metastases. Conversely, in the TKI+TRT group, 21 patients (30.4%) received radiotherapy for bone metastases and 19 patients (27.5%) for brain metastases.

### 3.2 Adding TRT improved survival outcomes

**Figure 1:**
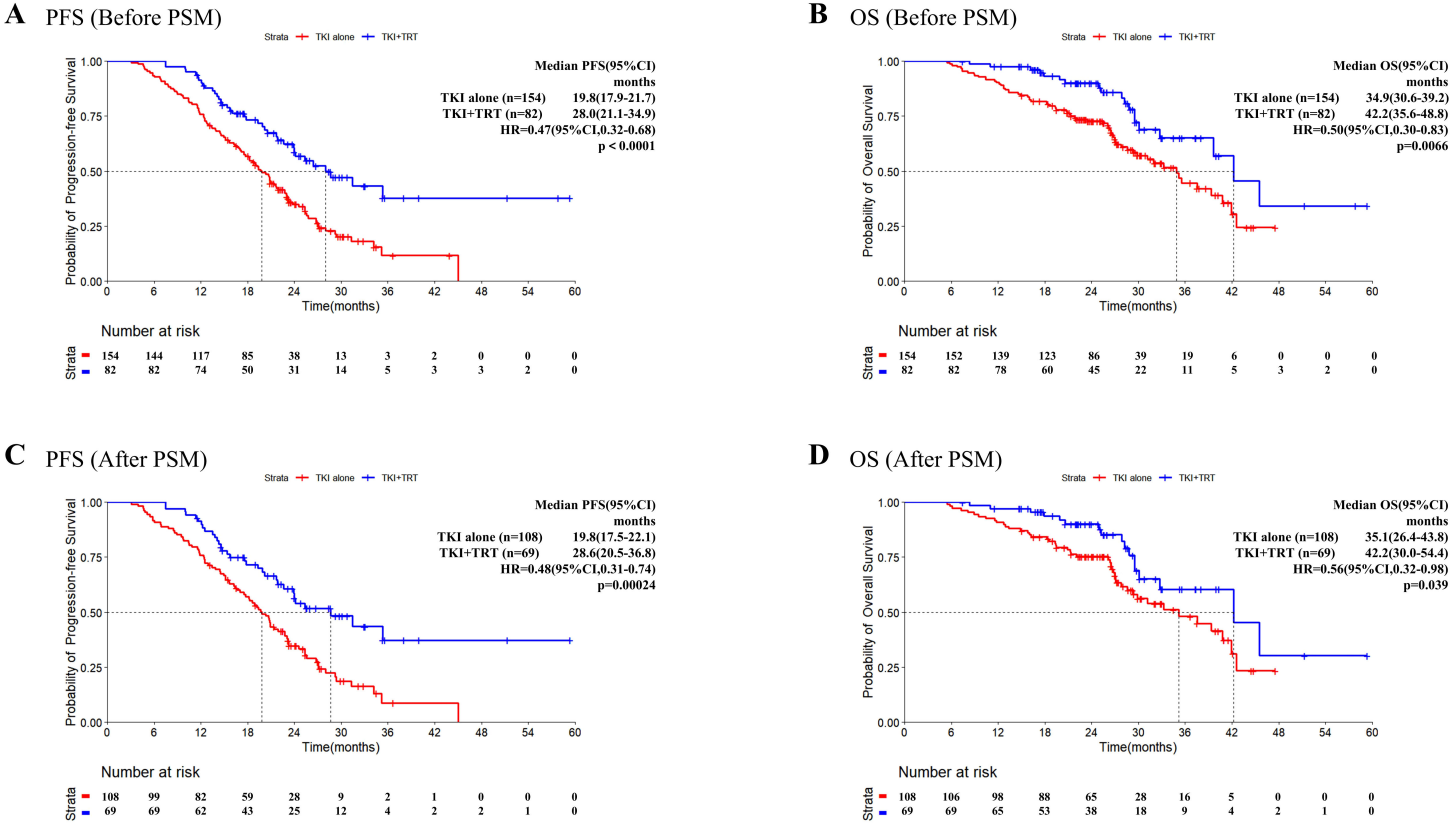
Progression-free survival and overall survival in patients before and after PSM. Kaplan–Meier survival analysis of PFS **(A)** and OS **(B)** of the TKI-alone and TKI+TRT groups before PSM, and Kaplan–Meier survival analysis of PFS **(C)** and OS **(D)** of the TKI-alone and TKI+TRT groups after PSM. OS, overall survival; PFS, progression-free survival; TKI, tyrosine kinase inhibitor; TRT, thoracic radiotherapy; PSM, propensity score matching.

For the total patient cohort, the median follow-up reached 28.4 months (range 11.5–59.4 months). Before PSM, patients in the TKI+TRT group had a median PFS of 28.0 months compared with 19.8 months for those in the TKI-alone group (**Figure 1A**). The median OS reached 34.9 months in the TKI-alone group and 42.2 months in the TKI+TRT group (**Figure 1B**).

After PSM, death or disease development occurred in 32 (46.4%) patients in the TKI+TRT group and 82 (75.9%) patients in the TKI-alone group by the cutoff date. The overall data maturity was 64.4%. Kaplan–Meier survival curves demonstrated early separation, indicating that the addition of TRT resulted in superior efficacy. Notably, the median PFS was significantly longer in the TKI+TRT group (28.6 months) than in the TKI-alone group (19.8 months, HR = 0.48, 95% confidence interval [CI]: 0.31–0.74, P = 0.00024, **Figure 1C**). At 2 years, 56.3% (95% CI:44.9%–70.7%) of patients were progression-free in the TKI+TRT group. Conversely, 34.7% (95% CI:26.5%–45.4%) were progression-free in the TKI-alone group.

After PSM, 63 patients died (17 and 46 in the TKI+TRT and TKI-alone groups, respectively). Overall data maturity was 35.6%. The median OS was significantly longer in the TKI+TRT group than in the TKI-alone group (42.2 months vs. 35.1 months, HR = 0.56, 95% CI: 0.32–0.98, P = 0.039, **Figure 1D**). The OS rate was 90.1% (95% CI: 82.8%–98.0%) at 2 years and 60.4% (95% CI: 45.4%–80.4%) at 3 years in the TKI+TRT group. In the TKI-alone group, the OS rate reached 75.3% (95% CI: 67.5%–84.1%) at 2 years and 48.1% (95% CI: 36.8%–62.9%) at 3 years.

According to multivariate Cox regression analyzes, Clinical N stage, EGFR mutation, TKI Response, and TRT were independent prognostic factors for PFS. Further, the analyses showed that age, number of metastatic organs, TKI Response, and TRT were independent prognostic factors for OS (**Supplementary Table 3**).

We further analyzed the progression pattern in each group. Before the cutoff date, 114 patients developed disease progression. 19 patients experienced progression from unknown causes or died, while detailed follow-up information was available for 95 patients, allowing for the analysis of their recurrence patterns. In the TKI+TRT group (n=28), progression of the primary disease was observed in three (10.7%) patients, metastatic site progression or new distant site progression in 23 (82.1%) patients, and progression of both sites in 2 (7.1%) patients. In the TKI-alone group (n=67), 17 (25.4%) patients had primary disease progression, 39 (58.2%) patients had metastatic site progression or new distant site progression, and 11 (16.4%) patients experienced progression of both sites.

### 3.3 Subgroup analysis of survival outcomes

**Figure 2:**
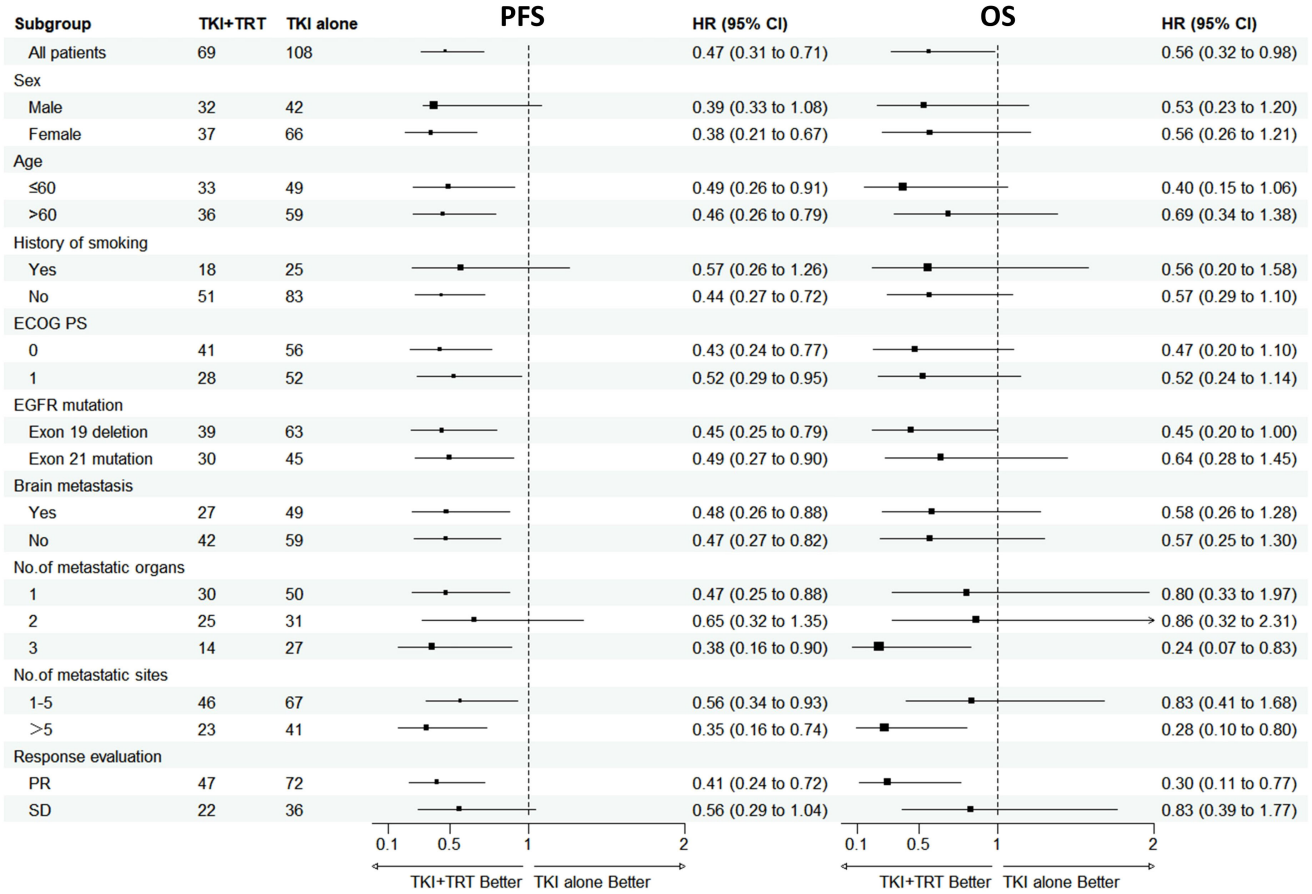
Subgroup analysis of progression-free survival and overall survival in patients after PSM. OS, overall survival; PFS, progression-free survival; ECOG PS, Eastern Cooperative Oncology Group performance status; EGFR, epidermal growth factor receptor; TKI, tyrosine kinase inhibitor; TRT, thoracic radiotherapy; PR, partial response; SD, stable disease.

An exploratory subgroup analysis was performed after PSM, with the results displayed in **Figure 2**. Compared with TKI alone, the PFS benefit provided by TKI+TRT seemed to remain uniform across various patient subgroups. In the subgroups of patients with exon 19 deletion, with the number metastatic organs=3, the with the number metastatic sites>5, and achieved PR to TKIs, the OS of TKI+TRT were more significant.

### 3.4 Optimal timing and doses of TRT

Univariate Cox analysis revealed that EGFR mutation type, TKI treatment response prior to TRT initiation, BED of TRT, and tumor status at the time of TRT initiation were significantly related to PFS. According to multivariate Cox regression analyses, tumor status at the time of TRT initiation (P = 0.024) was independent prognostic factor for PFS in patients who received TRT (n=82, **Supplementary Table 4**). Further, Univariate Cox analysis revealed that age, EGFR mutation type, Brain radiotherapy, TKI treatment response prior to TRT initiation, BED of TRT, and tumor status at the time of TRT initiation were significantly related to OS. Multivariate Cox regression analyses revealed that BED of TRT (P = 0.034) was independent prognostic factor for OS.

**Figure 3:**
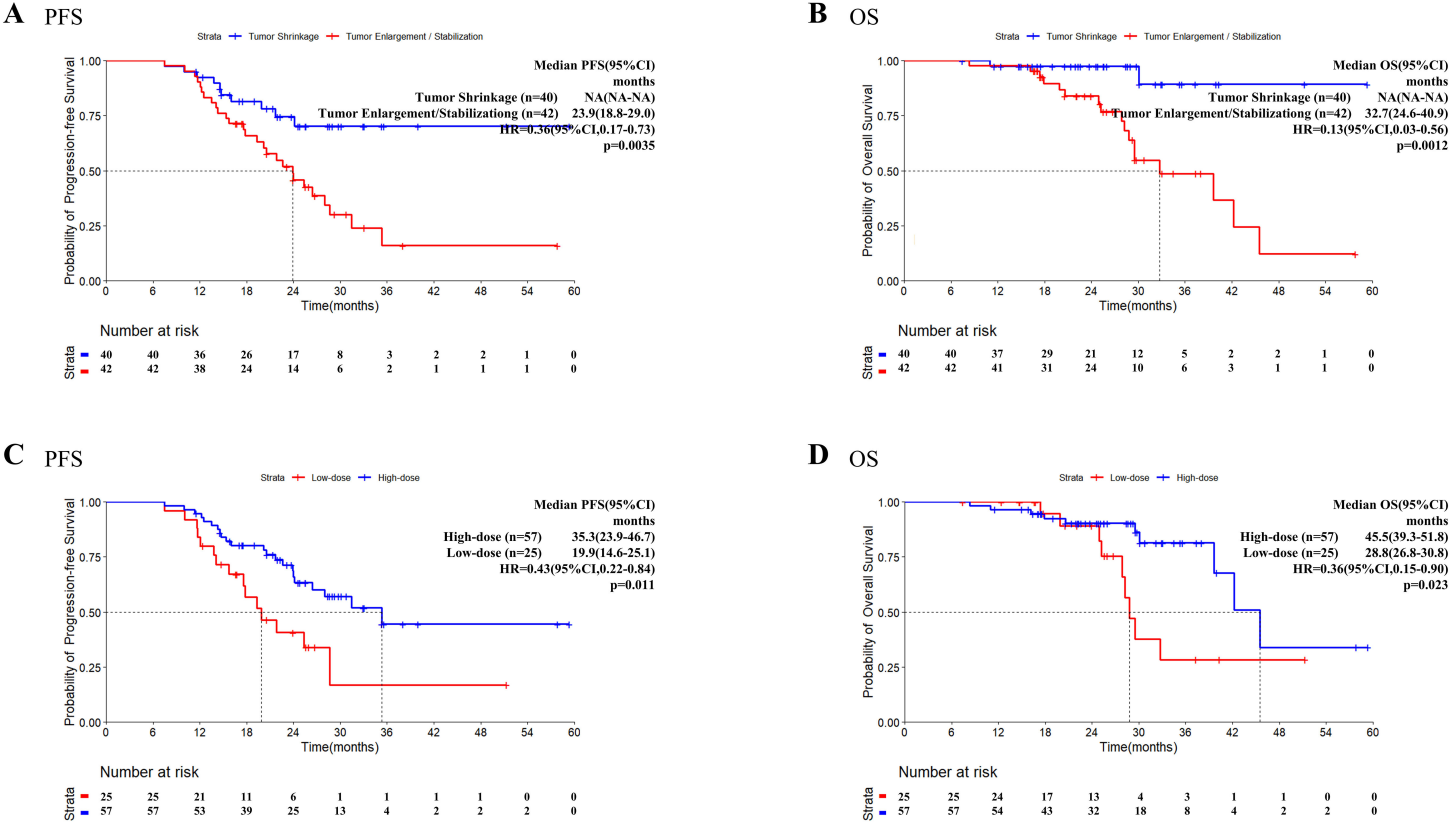
Progression-free survival and overall survival in patients who received TRT. Kaplan–Meier survival analysis of PFS **(A)** and OS **(B)** in patients with different tumor status at the time of TRT initiation. Kaplan–Meier survival analysis of PFS **(C)** and OS **(D)** in patients with high/low dose radiation. OS, overall survival; PFS, progression-free survival; TKI, tyrosine kinase inhibitor; TRT, thoracic radiotherapy.

We explored the influence of incorporating TRT into the treatment of tumors in states of enlargement/stabilization, or shrinkage on patient survival outcomes. Compared with the tumor enlargement/stabilization group, the tumor shrinkage group presented significantly better PFS (HR = 0.36, 95%CI: 0.17-0.73, P = 0.0035, **Figure 3A**) and better OS (HR = 0.13, 95%CI: 0.03-0.56, P = 0.0012, **Figure 3B**).

Information on the radiotherapy sites and doses for patients who received TRT before PSM is presented in **Supplementary Table 5**. Using the median BED of 60.0 Gy as the cutoff value, patients with a BED ≤ 60Gy were classified into the low-dose group (n=25) and patients with a BED>60Gy were classified into the high-dose group (n=57). Both the PFS (HR = 0.43, 95% CI: 0.22–0.84, P = 0.011, **Figure 3C**) and OS (HR = 0.36, 95% CI: 0.15–0.90, P = 0.023, **Figure 3D**) were significantly longer in the high-dose group than in the low-dose group.

### 3.5 Acceptable side effects

After PSM, the prevalence of acute (grade 3 or 4) TRAEs in the TKI+TRT group was elevated compared with that in the TKI-alone group (10.1% vs. 3.7%). The most common hematologic TRAEs in the TKI+TRT group were leukopenia (26.1%) and anemia (15.9%). In the TKI+TRT group, 15.9% of patients developed esophagitis, and 24.6% developed pneumonitis, exhibiting significantly higher incidences compared to those in the TKI-alone group (**Table 2**). However, only 5.8% of patients in the TKI+TRT group developed grade ≥3 pneumonitis.

**Table 2:** Treatment-related adverse events in the TKI-alone and TKI+TRT groups.

Characteristics	TKl alone group (n=108), No. (%)			TKI plus TRT group (n=69), No. (%)			P value
	Any grade	Grade 1/2	Grade 3/4	Any grade	Grade 1/2	Grade 3/4	
Esophagitis	2 (1.9)	2 (1.9)	0	11 (15.9)	10 (14.5)	1 (1.4)	<0.001
Pneumonitis	5 (4.6)	5 (4.6)	0	17 (24.6)	13 (18.8)	4 (5.8)	<0.001
Diarrhea	2 (1.9)	2 (1.9)	0	1 (1.4)	1 (1.4)	0	1.000
Vomiting	3 (2.8)	3 (2.8)	0	4 (5.8)	4 (5.8)	0	0.434
Decreased appetite	4 (3.7)	4 (3.7)	0	5 (7.2)	5 (7.2)	0	0.295
Dermatitis	1 (0.9)	1 (0.9)	0	3 (4.3)	3 (4.3)	0	0.301
Neutropenia	4 (3.7)	4 (3.7)	0	6 (8.7)	6 (8.7)	0	0.161
Leukopenia	25 (23.1)	23 (21.2)	2 (1.9)	18 (26.1)	17 (24.6)	1 (1.4)	0.657
Thrombocytopenia	15 (13.9)	14 (13.0)	1 (0.9)	11 (15.9)	10 (14.5)	1 (1.4)	0.707
Anemia	8 (7.4)	7 (6.5)	1 (0.9)	6 (8.7)	4 (5.8)	2 (2.9)	0.757
Elevated ALT and/or AST	4 (3.7)	4 (3.7)	0	5 (7.2)	5 (7.2)	0	0.295
Total	43 (39.8)	39 (36.1)	4 (3.7)	48 (69.6)	41 (59.4)	7 (10.1)	<0.001

TKI, tyrosine kinase inhibitor; TRT, thoracic radiotherapy. ALT, alanine aminotransferase, AST, aspartate aminotransferase.

## 4 Discussion

This study suggests that combining third-generation EGFR-TKI with TRT is associated with improved survival outcomes, both before and after PSM. Second, we investigated factors affecting the clinical outcomes of combined therapy, such as the optimal timing and dose of radiotherapy integration. Our data suggest that incorporating TRT during a phase of tumor shrinkage might be associated with optimal outcomes. Regarding the dose of TRT, our exploratory analysis indicated that higher BED were associated with longer PFS and OS in this cohort. Lastly, we determined that the combined therapy had a manageable safety profile, with toxicities that were consistent with the known profiles of each modality.

In contrast to conventional oligometastasis concepts, our study specifically focused on patients with oligo-organ metastasis (involving ≤3 metastatic organs without restrictions on metastatic site).Univariate Cox regression analysis revealed that the number of metastatic organs correlated with OS, whereas the number of metastatic sites was not significantly associated with PFS or OS. Additionally, subgroup analysis demonstrated that patients with more than five metastatic sites still had survival benefits from TRT. These findings suggest that, compared to metastases involving different organs, the number of sites within the same organ or region may be less clinically significant. Furthermore, the observed proportion of patients with oligo-organ metastases exceeds that of patients with oligometastatic. This finding suggests that TRT may result in a survival benefit in a larger population. The value of combining TRT with third-generation TKI in patients with Oligo-Organ Metastatic NSCLC remains unexplored. Our retrospective, real-world investigation was therefore conducted to offer additional evidence for this clinical question.

The addition of TRT may improve the local control rate (15). We explored patterns of progression and found that the rate of progression at the primary site decreased (10.7% vs. 25.4%) with the addition of TRT. In previous studies, targeted agents were shown to increase radiosensitivity and radiotherapy to reduce EGFR-TKI resistance. Kriegs and colleagues have demonstrated that in cells with intact p53/p21 signaling, the addition of EGFR inhibitors to radiotherapy resulted in the permanent blockade of the G1 phase of the cell cycle, which in turn enhanced cellular radiosensitivity (16). Chinnaiyan et al. reported that the combination of erlotinib and radiotherapy results in a further reduction in the proportion of cells in the S phase of the cell cycle. Erlotinib has been demonstrated to inhibit EGFR autophosphorylation and Rad51 expression, thereby increasing radiosensitivity (17). The aforementioned studies have identified the underlying cellular mechanisms, thereby providing theoretical support for combination therapy. However, the exact mechanism of the survival benefit of adding TRT to third-generation TKIs requires further investigation.

Only seven patients in this study received SBRT, whereas the others received IMRT. COX regression analyses demonstrated that the modality of radiotherapy did not constitute a significant factor in survival outcomes. COX regression analyses demonstrated that the modality of radiotherapy was not a significant factor in PFS and OS.

The dose and fractionation patterns of radiotherapy also differed. Therefore, we used the BED to calculate the dose received at the focal point of the lung tumor in patients in the TKI+TRT group to translate the various TRT segmentation patterns into a uniform dose. In one phase II clinical trial, the radiotherapy dose was 54–60 Gy (18). In the SINDAS clinical trial, the total dose to the radiotherapy group was 25–40 Gy (8). In another phase II clinical study, the radiotherapy modality was SBRT, with a dose ranging from 30–50 Gy (19). Despite the variation in dose and radiotherapy modality between these studies, all demonstrated a favorable survival benefit. As a retrospective study, we observed variations in radiotherapy doses prescribed by clinicians. Consequently, we compared the administered doses and found that higher doses may be associated with better outcomes in our cohort. However, further prospective or retrospective studies with larger sample sizes are required to provide robust evidence for guiding clinical decision-making.

The study by Yang et al. demonstrated that patients harboring exon 19 deletions or exon 21 L858R mutations exhibit distinct survival benefits following TKI treatment (20). Patients with exon 21 mutations have worse outcomes compared to those with exon 19 deletions following treatment with EGFR-TKIs (21, 22). An analysis of subgroups indicated that the PFS of the TKI+TRT group was improved among patients with exon 19 deletions and exon 21 mutations. This suggests that tailored combination therapy should be considered for patients with exon 21 mutations.

In the study by Zhang et al., patients were stratified into high- and low-regression groups based on a median maximal tumor reduction of 22%, demonstrating no significant differences in both PFS and OS (23). In contrast to their study, we stratified patients based on tumor regression versus no regression, revealing significant differences in both PFS and OS. A study has shown that initiating radiotherapy following PR is more effective than initiating radiotherapy during SD (24). Our findings further suggest that combined radiotherapy should be administered during tumor regression rather than enlargement. This may indicate that integrating TRT during the period when TKI is exerting its therapeutic effect could lead to a superior synergistic effect. Therefore, determining the optimal time to administer radiotherapy is important. In a previous study, Li and colleagues demonstrated that the mean tumor volume was significantly reduced within 40 days after treatment with EGFR-TKIs (25). Wu et al. reported a median response time of 7.4 weeks among responders following TKI treatment (26).This may represent an opportune timing for incorporating TRT. Certainly, the optimal timing for TRT remains to be determined in prospective studies.

Although treatment with TKI+TRT was significantly more effective than treatment with TKI alone, the prevalence of grade 3 TRAEs increased following this treatment. Studies have also shown the greater toxicity of TKI+TRT compared to that of TKI alone (9, 27).Radiation-related adverse events emerged post-radiotherapy initiation but were generally tolerable. Our previous study reported that only 5.1% (3/59) of patients treated with icotinib+TRT developed grade ≥3 radiation pneumonitis (RP), which suggests that the combination with radiotherapy is safe (9). Jia et al. reported that osimertinib combined with TRT resulted in the occurrence of grade ≥2 RP in seven out of 11 cases (63.6%) and grade 3 RP in five out of 11 cases (45.4%) (28). However, it is important to note that osimertinib was employed as second-line therapy in this study, and all patients exhibited resistance to first-line TKI therapy combined with a T790M mutation, which may explain the discrepancy with our conclusions. The potential risks associated with RP must be carefully considered, with particular attention to mean lung dose and V20.

However, our research still has some limitations. First, due to its retrospective design, we could not systematically collect and analyze co-mutations (such as cMET amplification and TP53 mutation) or more detailed tumor burden metrics (including the number of lymph node stations involved and specific lesion sizes). These factors are known to be closely associated with TKI efficacy and survival outcomes, and their uneven distribution may introduce residual confounding. Furthermore, we acknowledge that the ability to tolerate TRT may reflect a better underlying health status. Despite the use of PSM, unmeasured confounders, such as lesion size, location, vascular proximity, organ function, or subtle differences in performance status, could have influenced the outcomes. Lastly, this was a single-center retrospective analysis with inherent selection bias despite using PSM methods. The insufficient follow-up duration resulted in immature OS data for the TKI+TRT group. Additionally, post-progression treatment strategies were not analyzed. While this limitation may potentially impact OS outcomes, it does not affect the PFS results reported herein.

## 5 Conclusion

In conclusion, in the context of first-line therapy for patients with EGFR-mutated oligo-organ metastatic NSCLC, third-generation EGFR TKI+TRT enhanced PFS and OS more prominently than did EGFR TKI alone. The administration of higher radiation doses during a phase of tumor shrinkage may be associated with optimal outcomes. Although radiotoxicity and hematotoxicity occurred, these effects were consistent with the use of radiotherapy and the known safety profiles of individual agents and were tolerable in most patients.

## Data Availability

The raw data supporting the conclusions of this article will be made available by the authors, without undue reservation.

## Supplementary material

Supplementary Table 1 Metastasis in patients at baseline after PSM.

Supplementary Table 2 Radiotherapy sites in patients after PSM.

Supplementary Table 3 Cox regression analysis of PFS and OS in patients after PSM.

Supplementary Table 4 Cox regression analysis of PFS and OS in all patients who received TRT.

Supplementary Table 5 Radiotherapy sites and doses in patients who received TRT before PSM.

Supplementary Figure 1 Flow chart of the study. ECOG PS, Eastern Cooperative Oncology Group performance status; EGFR, epidermal growth factor receptor; PSM, propensity score matching; TKI, tyrosine kinase inhibitor; TRT, thoracic radiotherapy

## References

[1] D’Addario G Früh M Reck M Baumann P Klepetko W Felip E . FM. Metastatic non-small-cell lung cancer: ESMO Clinical Practice Guidelines for diagnosis, treatment and follow-up. Ann Oncol. (2010) 21:v116–9. doi: 10.1093/annonc/mdq189, PMID: 20555059

[2] Siegel RL Miller KD Fuchs HE Jemal A . Cancer statistics, 2021. CA Cancer J Clin. (2021) 71:7–33. doi: 10.3322/caac.21654, PMID: 33433946

[3] Sequist LV Bell DW Lynch TJ Haber DA . Molecular predictors of response to epidermal growth factor receptor antagonists in non-small-cell lung cancer. J Clin Oncol. (2007) 25:587–95. doi: 10.1200/JCO.2006.07.3585, PMID: 17290067

[4] Ettinger DS Wood DE Aisner DL Akerley W Bauman JR Bharat A . NCCN guidelines insights: non-small cell lung cancer, version 2. 2021. J Natl Compr Can Netw. (2021) 19:254–66. doi: 10.6004/jnccn.2021.0013, PMID: 33668021

[5] Al-Halabi H Sayegh K Digamurthy SR Niemierko A Piotrowska Z Willers H . Pattern of failure analysis in metastatic EGFR-mutant lung cancer treated with tyrosine kinase inhibitors to identify candidates for consolidation stereotactic body radiation therapy. J Thorac Oncol. (2015) 10:1601–7. doi: 10.1097/JTO.0000000000000648, PMID: 26313684

[6] Kong FM Zhao J Wang J Faivre-Finn C . Radiation dose effect in locally advanced non-small cell lung cancer. J Thorac Dis. (2014) 6:336–47. doi: 10.3978/j.issn.2072-1439.2014.01.23, PMID: 24688778 PMC3968556

[7] Gomez DR Tang C Zhang J Blumenschein GR Hernandez M Lee JJ . Local consolidative therapy vs. maintenance therapy or observation for patients with oligometastatic non-small-cell lung cancer: long-term results of a multi-institutional, Phase II, randomized study. J Clin Oncol. (2019) 37:1558–65. doi: 10.1200/JCO.19.00201, PMID: 31067138 PMC6599408

[8] Wang XS Bai YF VetV V RL YU Tian W Ao R . Randomized trial of first-line tyrosine kinase inhibitor with or without radiotherapy for synchronous oligometastatic EGFR-mutated non-small cell lung cancer. J Natl Cancer Inst. (2023) 115:742–8. doi: 10.1093/jnci/djac015, PMID: 35094066 PMC10248839

[9] Sun H Li M Huang W Zhang J Wei S Yang Y . Thoracic radiotherapy improves the survival in patients with EGFR-mutated oligo-organ metastatic non-small cell lung cancer treated with epidermal growth factor receptor-tyrosine kinase inhibitors: a multicenter, randomized, controlled, phase III trial. J Clin Oncol. (2025) 43:412–21. doi: 10.1200/JCO.23.02075, PMID: 39374473

[10] Soria JC Ohe Y Vansteenkiste J Reungwetwattana T Chewaskulyong B Lee KH . Osimertinib in untreated EGFR-mutated advanced non-small-cell lung cancer. N Engl J Med. (2018) 378:113–25. doi: 10.1056/NEJMoa1713137, PMID: 29151359

[11] Lu S Dong X Jian H Chen J Chen G Sun Y . AENEAS: a randomized phase III trial of aumolertinib versus gefitinib as first-line therapy for locally advanced or metastaticNon-small-cell lung cancer with EGFR exon 19 deletion or L858R mutations. J Clin Oncol. (2022) 40:3162–71. doi: 10.1200/JCO.21.02641, PMID: 35580297 PMC9509093

[12] Shi Y Chen G Wang X Liu Y Wu L Hao Y . Furmonertinib (AST2818) versus gefitinib as first-line therapy for Chinese patients with locally advanced or metastatic EGFR mutation-positive non-small-cell lung cancer (FURLONG): a multicentre, double-blind, randomised phase 3 study. Lancet Respir Med. (2022) 10:1019–28. doi: 10.1016/S2213-2600(22)00168-0, PMID: 35662408

[13] Fowler JF . The linear-quadratic formula and progress in fractionated radiotherapy. Br J Radiol. (1989) 62:679–94. doi: 10.1259/0007-1285-62-740-679, PMID: 2670032

[14] Austin PC . An introduction to propensity score methods for reducing the effects of confounding in observational studies. Multivariate Behav Res. (2011) 46:399–424. doi: 10.1080/00273171.2011.568786, PMID: 21818162 PMC3144483

[15] Wang J Xia TY Wang YJ Li HQ Li P Wang JD . Prospective study of epidermal growth factor receptor tyrosine kinase inhibitors concurrent with individualized radiotherapy for patients with locally advanced or metastatic non-small-cell lung cancer. Int J Radiat Oncol Biol Phys. (2011) 81:e59–65. doi: 10.1016/j.ijrobp.2010.12.035, PMID: 21345607

[16] Kriegs M Gurtner K Can Y Brammer I Rieckmann T Oertel R . Radiosensitization of NSCLC cells by EGFR inhibition is the result of an enhanced p53-dependent G1 arrest. Radiother Oncol. (2015) 115:120–7. doi: 10.1016/j.radonc.2015.02.018, PMID: 25796091

[17] Chinnaiyan P Huang S Vallabhaneni G Armstrong E Varambally S Tomlins SA . Mechanisms of enhanced radiation response following epidermal growth factor receptor signaling inhibition by erlotinib (Tarceva). Cancer Res. (2005) 65:3328–35. doi: 10.1158/0008-5472.CAN-04-3547, PMID: 15833866

[18] Zheng L Wang Y Xu Z Yang Q Zhu G Liao XY . Concurrent EGFR-TKI and thoracic radiotherapy as first-line treatment for stage IV non-small cell lung cancer harboring EGFR active mutations. Oncologist. (2019) 24:1031–e612. doi: 10.1634/theoncologist.2019-0285, PMID: 31040256 PMC6693693

[19] Peng P Gong J Zhang Y Zhou S Li Y Han G . EGFR-TKIs plus stereotactic body radiation therapy (SBRT) for stage IV non-small cell lung cancer (NSCLC): A prospective, multicenter, randomized, controlled phase II study. Radiother Oncol. (2023) 184:109681. doi: 10.1016/j.radonc.2023.109681, PMID: 37105304

[20] Yang JC Wu YL Schuler M Sebastian M Popat S Yamamoto N . Afatinib versus cisplatin-based chemotherapy for EGFR mutation-positive lung adenocarcinoma (LUX-Lung 3 and LUX-Lung 6): analysis of overall survival data from two randomised, phase 3 trials. Lancet Oncol. (2015) 16:141–51. doi: 10.1016/S1470-2045(14)71173-8, PMID: 25589191

[21] Planchard D Jänne PA Cheng Y Yang JC Yanagitani N Kim SW . Osimertinib with or without chemotherapy in EGFR-mutated advanced NSCLC. N Engl J Med. (2023) 389:1935–48. doi: 10.1056/NEJMoa2306434, PMID: 37937763

[22] Lee CK Davies L Wu YL Mitsudomi T Inoue A Rosell R . Gefitinib or erlotinib vs chemotherapy for EGFR mutation-positive lung cancer: individual patient data meta-analysis of overall survival. J Natl Cancer Inst. (2017) 109(6):djw279. doi: 10.1093/jnci/djw279, PMID: 28376144

[23] Zhang J Huang Y Li X Guo Y Zhao Y Xue C . The impact of tumor size change after target therapy on survival: analysis of patients enrolled onto three clinical trials of advanced NSCLC from one institution. Onco Targets Ther. (2012) 5:349–55. doi: 10.2147/OTT.S38441, PMID: 23172990 PMC3501954

[24] Tang Y Xia B Xie R Xu X Zhang M Wu K . Timing in combination with radiotherapy and patterns of disease progression in non-small cell lung cancer treated with EGFR-TKI. Lung Can. (2020) 140:65–70. doi: 10.1016/j.lungcan.2019.12.009, PMID: 31884128

[25] Li Q Liang N Zhang X Zhang Y Ouyang W Su S . Reasonable timing of radiotherapy for stage IV non-small-cell lung cancer during targeted therapy based on tumour volume change. Front Oncol. (2021) 11:705303. doi: 10.3389/fonc.2021.705303, PMID: 34631535 PMC8496348

[26] Wu TH Hsiue EH Lee JH Lin CC Liao WY Ho CC . Best response according to RECIST during first-line EGFR-TKI treatment predicts survival in EGFR mutation-positive non-small-cell lung cancer patients. Clin Lung Can. (2018) 19:e361–72. doi: 10.1016/j.cllc.2018.01.005, PMID: 29477365

[27] Wang X Zeng Z Cai J Xu P Liang P Luo Y . Efficacy and acquired resistance for EGFR-TKI plus thoracic SBRT in patients with advanced EGFR-mutant non-small-cell lung cancer: a propensity-matched retrospective study. BMC Can. (2021) 21:482. doi: 10.1186/s12885-021-08228-2, PMID: 33931014 PMC8086057

[28] Jia W Guo H Jing W Jing X Li J Wang M . An especially high rate of radiation pneumonitis observed in patients treated with thoracic radiotherapy and simultaneous osimertinib. Radiother Oncol. (2020) 152:96–100. doi: 10.1016/j.radonc.2020.07.051, PMID: 32745669

