# Supplementary material for: Effect, safety, timing and dose of thoracic radiotherapy plus third-generation EGFR-TKIs as first-line treatment in patients with EGFR-mutated oligo-organ metastatic NSCLC
Source: Front Oncol. 2025 Nov 26;15:1707876. doi: 10.3389/fonc.2025.1707876 (PMC12689311; doi:10.3389/fonc.2025.1707876)
Supplement: Supplementary Table 1 — Metastasis in patients at baseline after PSM. [file DataSheet1.docx]

**List of Supporting Information**:

**Supplementary Table 1.** **Metastasis in patients at baseline after PSM**

**Supplementary Table 2. Radiotherapy sites in patients after PSM**

**Supplementary Table 3. Cox regression analysis of PFS and OS in patients after PSM**

**Supplementary Table 4. Cox regression analysis of PFS and OS in all patients who received TRT**

**Supplementary Table 5. Radiotherapy sites and doses in patients who received TRT before PSM**

**Supplementary Figure 1. Flow chart of the study**

**Supplementary Table 1. Metastasis in patients at baseline after PSM**

| Metastatic Organs | After PSM | |  |
| --- | --- | --- | --- |
|  | TKI alone  （n=108,%） | TKI+TRT  （n=69,%） | *P value* |
| Lung metastasis |  |  | 0.597 |
| NO | 71 (65.7%) | 48 (69.6%) |  |
| YES | 37 (34.3%) | 21 (30.4%) |  |
| Brain metastasis |  |  | 0.508 |
| NO | 59 (54.6%) | 42 (60.9%) |  |
| YES | 49 (45.4%) | 27 (39.1%) |  |
| Bone metastasis |  |  | 0.749 |
| NO | 49 (45.4%) | 33 (47.8%) |  |
| YES | 59 (54.6%) | 36 (52.2%) |  |
| Liver metastasis |  |  | 1.000 |
| NO | 104 (96.3%) | 67 (97.1%) |  |
| YES | 4 (3.7%) | 2 (2.9%) |  |
| Adrenal gland metastasis |  |  | 0.528 |
| NO | 99 (91.7%) | 65 (94.2%) |  |
| YES | 9 (8.3%) | 4 (5.8%) |  |
| Pleural metastasis |  |  | 0.053 |
| NO | 82 (75.9%) | 43 (62.3%) |  |
| YES | 26 (24.1%) | 26 (37.7%) |  |
| Others |  |  | 0.854 |
| NO | 101 (93.5%) | 65 (94.2%) |  |
| YES | 7 (6.5%) | 4 (5.8%) |  |

Abbreviations: PSM, propensity score matching; TKI, tyrosine kinase inhibitor; TRT, thoracic radiotherapy.

**Supplementary Table 2. Radiotherapy sites** **in patients after PSM**

|  | | TKI alone | TKI+TRT |
| --- | --- | --- | --- |
| **Total patients** | | **108** | **69** |
| **Radiotherapy of thoracic** | | **0** | **69** |
| Dose | BED(10) |  |  |
| IMRT: 45Gy(3Gy/F×15F) | 58.5 Gy | 0 | 9 |
| 50Gy(5Gy/F×10F) | 75 Gy | 0 | 2 |
| 50Gy(2.5Gy/F×20F) | 62.5 Gy | 0 | 3 |
| 50Gy(2Gy/F×25F) | 60 Gy | 0 | 13 |
| 51Gy(3Gy/F×17F) | 66.3 Gy | 0 | 3 |
| 54Gy(3Gy/F×18F) | 70.2 Gy | 0 | 3 |
| 54Gy(2Gy/F×27F) | 64.8 Gy | 0 | 8 |
| 55Gy(2.2Gy/F×25F) | 67.1 Gy | 0 | 2 |
| 56Gy(2Gy/F×28F) | 67.2 Gy | 0 | 6 |
| 58Gy(2Gy/F×29F) | 69.6 Gy | 0 | 1 |
| 59.4Gy(1.8Gy/F×33F) | 70.09 Gy | 0 | 1 |
| 60Gy(5Gy/F×12F) | 90 Gy | 0 | 1 |
| 60Gy(4Gy/F×15F) | 84 Gy | 0 | 1 |
| 60Gy(3Gy/F×20F) | 78 Gy | 0 | 3 |
| 60Gy(2Gy/F×30F) | 72 Gy | 0 | 4 |
| 66Gy(2Gy/F×33F) | 79.2 Gy | 0 | 1 |
| 70Gy(5Gy/F×28F) | 87.5 Gy | 0 | 1 |
| 75Gy(5Gy/F×15F) | 112.5 Gy | 0 | 1 |
| SBRT:50Gy(10Gy/F×5F) | 100 Gy | 0 | 1 |
| 56Gy(8Gy/F×7F) | 82.5 Gy | 0 | 4 |
| 55Gy(5Gy/F×11F | 100.8 Gy | 0 | 1 |
| **Radiotherapy of metastatic sites** | | **35** | **41** |
| **Radiotherapy of brain metastasis** | | **21** | **19** |
| Dose | |  |  |
| SBRT:50Gy(5Gy/F×10F) | | 0 | 1 |
| 27Gy(9Gy/F×3F) | | 0 | 1 |
| Cybeknife: 36Gy(12Gy/F×3F) | | 0 | 1 |
| Ganma knife: 20Gy(20Gy/F×1F) | | 0 | 1 |
| IMRT: 30Gy-40Gy | | 9 | 2 |
| ＞40Gy-50Gy | | 10 | 8 |
| ＞50Gy-60Gy | | 2 | 5 |
| **Radiotherapy of bone metastasis** | | **15** | **21** |
| Dose | |  |  |
| 30Gy-40Gy | | 14 | 18 |
| ＞40Gy-50Gy | | 1 | 2 |
| ＞50Gy-60Gy | | 0 | 1 |
| **Radiotherapy of chest wall** | | **0** | **2** |
| Dose | |  |  |
| 50Gy(2Gy/F×25F) | | 0 | 1 |
| 60Gy(6Gy/F×10F) | | 0 | 1 |
| **Radiotherapy of cervical lymph node** | | **1** | **0** |
| Dose | |  |  |
| 56Gy(2Gy/F×28F) | | 1 | 0 |

Abbreviations:TKI, tyrosine kinase inhibitor; TRT, thoracic radiotherapy;IMRT, intensity-modulated radiotherapy;PSM, propensity score matching;SBRT,stereotactic body radiation therapy

**Supplementary Table3. Cox regression analysis of the PFS and OS in patients after PSM.**

| **Variables** | **PFS Univariate analysis** |  | **PFS Multivariate analysis** |  | **OS Univariate analysis** |  | **OS Multivariate analysis** |  |
| --- | --- | --- | --- | --- | --- | --- | --- | --- |
|  | **HR (95 %CI)** | ***P*** | **HR (95 %CI)** | ***P*** | **HR (95 %CI)** | ***P*** | **HR (95 %CI)** | ***P*** |
| **Age** |  |  |  |  |  |  |  |  |
| < 60 years | 0.732(0.503,1.063) | 0.102 |  |  | 0.514(0.307,0.862) | 0.012 | **0.527(0.307,0.905)** | **0.020** |
| ≥ 60 years | Reference |  |  |  | Reference |  | Reference |  |
| **Sex** |  |  |  |  |  |  |  |  |
| Female | 1.087(0.749,1.577) | 0.662 |  |  | 1.410(0.854,2.326) | 0.179 |  |  |
| Male | Reference |  |  |  | Reference |  |  |  |
| **ECOG PS** |  |  |  |  |  |  |  |  |
| 0 | 0.915(0.633,1.323) | 0.637 |  |  | 0.741(0.451,1.216) | 0.235 |  |  |
| 1 | Reference |  |  |  | Reference |  |  |  |
| **Smoking** |  |  |  |  |  |  |  |  |
| YES | Reference |  |  |  | Reference |  |  |  |
| NO | 1.043(0.680,1.600) | 0.845 |  |  | 0.813(0.465,1.420) | 0.467 |  |  |
| **Clinical T stage** |  |  |  |  |  |  |  |  |
| T1–2 | 0.916(0.611,1.375) | 0.673 |  |  | 0.955(0.547,1.668) | 0.872 |  |  |
| T3–4 | Reference |  |  |  | Reference |  |  |  |
| **Clinical N stage** |  |  |  |  |  |  |  |  |
| N0-1 | 0.573(0.353,0.930) | 0.024 | **0.492(0.302,0.803)** | **0.005** | 1.049(0.562,1.958) | 0.880 |  |  |
| N2-3 | Reference |  | Reference |  | Reference |  |  |  |
| **No. of metastatic organs** |  |  |  |  |  |  |  |  |
| 1 | 0.755(0.480,1.188) | 0.225 |  |  | 0.445(0.245,0.807) | 0.008 | **0.409(0.217,0.772)** | **0.006** |
| 2 | 0.709(0.433,1.160) | 0.171 |  |  | 0.556(0.299,1.036) | 0.064 | **0.438(0.228,0.842)** | **0.013** |
| 3 | Reference |  |  |  | Reference |  | Reference |  |
| **No. of metastatic sites** |  |  |  |  |  |  |  |  |
| 1-5 | 0.993(0.678,1.455) | 0.973 |  |  | 0.658(0.399,1.086) | 0.102 |  |  |
| ＞5 | Reference |  |  |  | Reference |  |  |  |
| **Bone metastasis** |  |  |  |  |  |  |  |  |
| YES | Reference |  |  |  | Reference |  |  |  |
| NO | 0.810(0.558,1.177) | 0.269 |  |  | 0.815(0.492,1.352) | 0.428 |  |  |
| **Liver metastasis** |  |  |  |  |  |  |  |  |
| YES | Reference |  |  |  | Reference |  |  |  |
| NO | 0.932(0.379,2.290) | 0.878 |  |  | 0.403(0.143,1.137) | 0.086 |  |  |
| **Brain metastasis** |  |  |  |  |  |  |  |  |
| YES | Reference |  |  |  | Reference |  | Reference |  |
| NO | 0.728(0.503,1.053) | 0.092 |  |  | 0.495(0.300,0.817) | 0.006 | 0.761(0.443,1.309) | 0.324 |
| **EGFR** **mutation** |  |  |  |  |  |  |  |  |
| Exon 19 deletion | 0.683(0.470,0.994) | 0.047 | **0.674(0.462,0.984)** | **0.041** | 0.675(0.406,1.123) | 0.130 |  |  |
| Exon 21 mutation | Reference |  | Reference |  | Reference |  |  |  |
| **Thoracic radiotherapy** |  |  |  |  |  |  |  |  |
| YES | Reference |  | Reference |  | Reference |  | Reference |  |
| NO | 2.124(1.407,3.205) | ＜0.001 | **2.279(1.505,3.450)** | **＜0.001** | 1.788(1.021,3.129) | 0.042 | **0.761(0.443,1.309)** | **0.034** |
| **Bone radiotherapy** |  |  |  |  |  |  |  |  |
| YES | Reference |  |  |  | Reference |  |  |  |
| NO | 0.905(0.580,1.412) | 0.661 |  |  | 0.785(0.438,1.408) | 0.417 |  |  |
| **Brain radiotherapy** |  |  |  |  |  |  |  |  |
| YES | Reference |  |  |  | Reference |  |  |  |
| NO | 0.775(0.501,1.198) | 0.252 |  |  | 0.593(0.338,1.038) | 0.067 |  |  |
| **Response evaluation*** |  |  |  |  |  |  |  |  |
| PR | 0.632(0.434,0.920) | 0.017 | **0.622(0.425,0.911)** | **0.015** | 0.531(0.323,0.874) | 0.013 | **0.470(0.279,0.794)** | **0.005** |
| SD | Reference |  | Reference |  | Reference |  | Reference |  |

Abbreviations: PSM,propensity score matching; ECOG PS, Eastern Cooperative Oncology Group performance status; EGFR, epidermal growth factor receptor;PR,partial response; SD, stable disease

*TKI treatment response prior to TRT initiation

**Supplementary Table 4. Cox regression analysis of the PFS and OS in all patients received TRT.**

| **Variables** | **PFS Univariate analysis** |  | **PFS Multivariate analysis** |  | **OS Univariate analysis** |  | **OS Multivariate analysis** |  |
| --- | --- | --- | --- | --- | --- | --- | --- | --- |
|  | **HR (95 %CI)** | ***P*** | **HR (95 %CI)** | ***P*** | **HR (95 %CI)** | ***P*** | **HR (95 %CI)** | ***P*** |
| **Age** |  |  |  |  |  |  |  |  |
| < 60 years | 0.738(0.385,1.141) | 0.360 |  |  | 0.250(0.089,0.703) | 0.009 | 0.410(0.123,1.374) | 0.149 |
| ≥ 60 years | Reference |  |  |  | Reference |  | Reference |  |
| **Sex** |  |  |  |  |  |  |  |  |
| Male | 1.260(0.659,2.412) | 0.484 |  |  | 0.999(0.405,2.465) | 0.999 |  |  |
| Female | Reference |  |  |  | Reference |  |  |  |
| **ECOG PS** |  |  |  |  |  |  |  |  |
| 0 | 0.738(0.386,1.410) | 0.358 |  |  | 0.532(0.212,1.331) | 0.177 |  |  |
| 1 | Reference |  |  |  | Reference |  |  |  |
| **Smoking** |  |  |  |  |  |  |  |  |
| YES | Reference |  |  |  | Reference |  |  |  |
| NO | 0.911(0.428,1.936) | 0.808 |  |  | 0.773(0.277,2.155) | 0.623 |  |  |
| **Clinical T stage** |  |  |  |  |  |  |  |  |
| T1–2 | 1.324(0.638,2.742) | 0.450 |  |  | 1.538(0.509,4.650) | 0.446 |  |  |
| T3–4 | Reference |  |  |  | Reference |  |  |  |
| **Clinical N stage** |  |  |  |  |  |  |  |  |
| N0-1 | 0.561(0.234,1.345) | 0.195 |  |  | 0.775(0.221,2.715) | 0.690 |  |  |
| N2-3 | Reference |  |  |  | Reference |  |  |  |
| **No. of metastatic organs** |  |  |  |  |  |  |  |  |
| 1 | 0.881(0.365,2.131) | 0.779 |  |  | 0.844(0.218,3.278) | 0.807 |  |  |
| 2 | 1.109(0.440,2.798) | 0.826 |  |  | 1.823(0.491,6.769) | 0.370 |  |  |
| 3 | Reference |  |  |  | Reference |  |  |  |
| **No. of metastatic sites** |  |  |  |  |  |  |  |  |
| 1-5 | 1.178(0.603,2.299) | 0.632 |  |  | 1.164(0.455,2.976) | 0.752 |  |  |
| ＞5 | Reference |  |  |  | Reference |  |  |  |
| **Bone metastasis** |  |  |  |  |  |  |  |  |
| YES | Reference |  |  |  | Reference |  |  |  |
| NO | 0.778(0.403,1.500) | 0.453 |  |  | 1.146(0.428,3.068) | 0.786 |  |  |
| **Liver metastasis** |  |  |  |  |  |  |  |  |
| YES | Reference |  |  |  | Reference |  |  |  |
| NO | 0.671(0.091,4.966) | 0.696 |  |  | 0.234(0.029,1.875) | 0.171 |  |  |
| **Brain metastasis** |  |  |  |  |  |  |  |  |
| YES | Reference |  |  |  | Reference |  |  |  |
| NO | 0.817(0.424,1.577) | 0.548 |  |  | 0.447(0.181,1.106) | 0.082 |  |  |
| **EGFR** **mutation** |  |  |  |  |  |  |  |  |
| Exon 19 deletion | 0.512(0.264,0.993) | 0.048 | 0.522(0.262,1.041) | 0.065 | 0.360(0.138,0.940) | 0.037 | 0.433(0.151,1.238) | 0.118 |
| Exon 21 mutation | Reference |  | Reference |  | Reference |  |  |  |
| **Bone radiotherapy** |  |  |  |  |  |  |  |  |
| YES | Reference |  |  |  | Reference |  |  |  |
| NO | 0.656(0.337,1.276) | 0.214 |  |  | 0.643(0.257,1.610) | 0.346 |  |  |
| **Brain radiotherapy** |  |  |  |  |  |  |  |  |
| YES | Reference |  |  |  | Reference |  |  |  |
| NO | 0.663(0.326,1.349) | 0.257 |  |  | 0.380(0.153,0.947) | 0.038 | 0.325(0.082,1.288) | 0.110 |
| **Radiotherapy Modality** |  |  |  |  |  |  |  |  |
| SBRT | Reference |  |  |  | Reference |  |  |  |
| IMRT | 1.720(0.519,5.704) | 0.375 |  |  | 1.411(0.319,6.237) | 0.650 |  |  |
| **Response evaluation*** |  |  |  |  |  |  |  |  |
| PR | 0.500(0.262,0.953) | 0.035 | 1.346(0.567,3.198) | 0.501 | 0.284(0.107,0.753) | 0.011 | 0.612(0.167,2.251) | 0.460 |
| SD | Reference |  | Reference |  | Reference |  | Reference |  |
| **BED** |  |  |  |  |  |  |  |  |
| ≤60 | 2.321(1.191,4.523) | 0.013 | 2.024(0.997,4.108) | 0.051 | 2.748(1.110,6.805) | 0.029 | **3.696(1.102,12.395)** | **0.034** |
| ＞60 | Reference |  | Reference |  | Reference |  | Reference |  |
| **Tumor status** |  |  |  |  |  |  |  |  |
| Grow/Stabilize | Reference |  | Reference |  | Reference |  | Reference |  |
| Shrink | 0.355(0.172,0.734) | 0.005 | **0.352(0.142,0.872)** | **0.024** | 0.129(0.030,0.560) | 0.006 | 0.264(0.044,1.588) | 0.146 |

Abbreviations: PSM,propensity score matching; ECOG PS, Eastern Cooperative Oncology Group performance status;

EGFR, epidermal growth factor receptor;SBRT , stereotactic body radiation therapy; IMRT , intensity‑modulated radiotherapy;

BED, biologically effective dose;PR,partial response; SD, stable disease

*TKI treatment response prior to TRT initiation

**Supplementary Table 5. Radiotherapy sites and doses in patients received TRT before PSM**

|  | | TKI+TRT |
| --- | --- | --- |
| **Total patients** | | **82** |
| **Radiotherapy of thoracic** | | **82** |
| Dose | BED(10) |  |
| IMRT: 30Gy(2.5Gy/F×12F) | 37.5 Gy | 1 |
| 30Gy(2Gy/F×15F) | 36 Gy | 1 |
| 45Gy(3Gy/F×15F) | 58.5 Gy | 9 |
| 48Gy(2Gy/F×24F) | 57.5 Gy | 1 |
| 50Gy(10Gy/F×5F) | 100 Gy | 1 |
| 50Gy(5Gy/F×10F) | 75 Gy | 3 |
| 50Gy(2.5Gy/F×20F) | 62.5 Gy | 4 |
| 50Gy(2Gy/F×25F) | 60 Gy | 13 |
| 51Gy(3Gy/F×17F) | 66.3 Gy | 3 |
| 54Gy(3Gy/F×18F) | 70.2 Gy | 3 |
| 54Gy(2Gy/F×27F) | 64.8 Gy | 8 |
| 55Gy(2.2Gy/F×25F) | 67.1 Gy | 3 |
| 56Gy(2Gy/F×28F) | 67.2 Gy | 7 |
| 58.5Gy(2.25Gy/F×26F) | 71.66 Gy | 1 |
| 58Gy(2Gy/F×29F) | 69.6 Gy | 1 |
| 59.4Gy(1.8Gy/F×33F) | 70.09 Gy | 1 |
| 60Gy(5Gy/F×12F) | 90 Gy | 1 |
| 60Gy(4Gy/F×15F) | 84 Gy | 1 |
| 60Gy(3Gy/F×20F) | 78 Gy | 1 |
| 60Gy(2Gy/F×30F) | 72 Gy | 8 |
| 66Gy(2Gy/F×33F) | 79.2 Gy | 1 |
| 70Gy(2.5Gy/F×28F) | 87.5 Gy | 1 |
| 75Gy(5Gy/F×15F) | 112.5 Gy | 2 |
| SBRT:48Gy(12Gy/F×4F) | 105.6 Gy | 1 |
| 50Gy(10Gy/F×5F) | 100 Gy | 1 |
| 55Gy(5Gy/F×11F) | 82.5 Gy | 1 |
| 56Gy(8Gy/F×7F) | 100.8 Gy | 4 |
| **Radiotherapy of metastatic sites** | | **45** |
| **Radiotherapy of brain metastasis** | | **21** |
| Dose | |  |
| SBRT:27Gy(9Gy/F×3F) | | 1 |
| 50Gy(5Gy×10F) | | 1 |
| Cybe knife: 36Gy(12Gy/F×3F) | | 1 |
| Ganma knife: 20Gy(20Gy/F×1F) | | 1 |
| IMRT: 30Gy-40Gy | | 2 |
| ＞40Gy-50Gy | | 10 |
| ＞50Gy-60Gy | | 5 |
| **Radiotherapy of bone metastasis** | | **24** |
| Dose | |  |
| 30Gy-40Gy | | 21 |
| ＞40Gy-50Gy | | 2 |
| ＞50Gy-60Gy | | 1 |
| **Radiotherapy of chest wall** | | **2** |
| Dose | |  |
| 50Gy(2Gy/F×25F) | | 1 |
| 60Gy(6Gy/F×10F) | | 1 |

Abbreviations:TKI, tyrosine kinase inhibitor; TRT, thoracic radiotherapy;IMRT, intensity-modulated radiotherapy;PSM, propensity score matching;SBRT,stereotactic body radiation therapy.

**Supplementary Figure 1.**

**
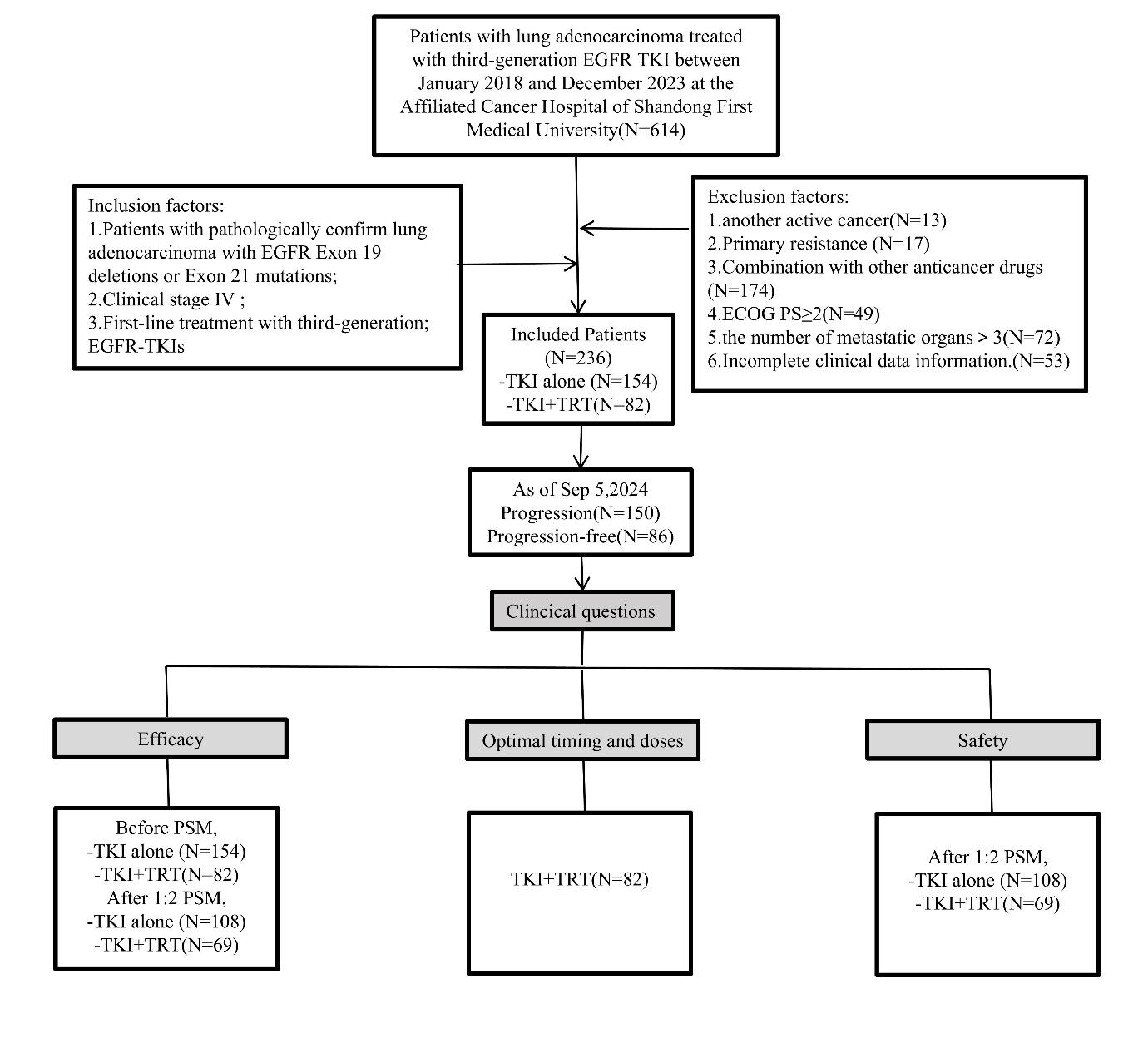
**

**Supplementary Figure 1. Flow chart of the study**

ECOG PS, Eastern Cooperative Oncology Group performance status; EGFR, epidermal growth factor receptor; PSM, propensity score matching; TKI, tyrosine kinase inhibitor; TRT, thoracic radiotherapy
